# Treatment of pulmonary arterial hypertension in congenital heart disease in Singapore versus the Netherlands: age exceeds ethnicity in influencing clinical outcome

**DOI:** 10.1007/s12471-016-0820-z

**Published:** 2016-03-17

**Authors:** A. C. M. J. van Riel, M. J. Schuuring, I. D. van Hessen, A. P. J. van Dijk, E. S. Hoendermis, J. W. Yip, B. J. M. Mulder, B. J. Bouma

**Affiliations:** 1 Department of Cardiology, Academic Medical Centre, Amsterdam, The Netherlands; 2 ICIN – Netherlands Heart Institute, Utrecht, The Netherlands; 3 Department of Cardiology, Radboud University Medical Centre, Nijmegen, The Netherlands; 4 Department of Cardiology, University Medical Centre Groningen, Groningen, The Netherlands; 5 Department of Cardiology, National University Heart Centre, Singapore, Singapore

**Keywords:** Pulmonary arterial hypertension, Congenital heart disease, Advanced treatment, Six minute walk test, Clinical outcome

## Abstract

**Background:**

Advanced treatment of pulmonary arterial hypertension (PAH) in congenital heart disease (CHD) is increasingly applied worldwide following the—mainly Western world based—international PAH-CHD guidelines. However, studies comparing clinical presentation and outcome after the initiation of PAH-specific treatment are lacking. We aimed to analyse this in a Singaporean and Dutch cohort of PAH-CHD patients.

**Methods:**

Adult CHD patients starting PAH-specific therapy, enrolled in two nationwide registries, were analysed. Patients received phosphodiesterase-type-5 inhibitors, endothelin receptor antagonists, or a combination. Change in six-minute walk test (6MWT) during follow-up was analysed using linear mixed model analysis. Determinants for mortality were assessed using Cox proportional hazard analyses.

**Results:**

A total of 74 patients, 45 Dutch (mean age 47 ± 14 years) and 29 Singaporean (mean age 41 ± 14 years) were analysed. Despite a lower 6MWT (312 versus 395 metres, *p* = 0.01) and peak VO_2_ (35 versus 49 % of predicted, *p* = 0.01) at baseline in Singaporean patients, the treatment effect was similar in the two populations. Age at initiation of therapy (per 5 year lower age, β = + 4.5, *p* = 0.017) was the strongest predictor of improvement in exercise capacity, corrected for ethnicity, baseline 6MWT, sex and CHD defect.

**Conclusions:**

Patients from Singapore had a worse clinical performance at baseline compared with the PAH-CHD patients from the Netherlands. No relation between ethnicity and improvement in 6MWT after PAH-specific therapy was found. Age at initiation of PAH-specific therapy was the strongest predictor of treatment efficacy and mortality, emphasising the need for early initiation of treatment in these patients.

## Introduction

Patients with congenital heart disease (CHD) may suffer from pulmonary arterial hypertension (PAH), leading to increased morbidity and mortality [1]. With the emergence of disease-targeting therapies, including prostanoids, endothelin receptor antagonists (ERAs) and phosphodiesterase-5 (PDE-5) inhibitors, it has become possible to improve symptoms and stabilise disease progression [2–4]. Current European Society of Cardiology guidelines recommend both ERAs and PDE-5 inhibitors as a class I-A indication for therapy in PAH patients in New York Heart Association (NYHA) functional class II or III [5]. Following these guidelines, PAH-specific treatment in CHD is increasingly applied worldwide [6]. However, these guidelines are based on randomised clinical trials which include only a small portion of CHD patients [7–9]. Furthermore, only 6–7 % of the patient cohort included in these trials is of Asian ethnicity, although the general Asian population accounts for more than 60 % of the total world population [10]. While the influence of ethnicity on CHD prevalence, clinical presentation and outcomes has been described [11, 12], studies regarding ethnicity and geographical variations on treatment outcome are sparse. However, biological differences are known to exist in the production and handling of ET-1, a potent systemic and pulmonary vasoconstrictor [13, 14]. Furthermore, socioeconomic factors and differences in healthcare systems could influence the effect of treatment in PAH-CHD [15]. We aimed to evaluate variations in clinical presentation and outcome after initiation of PAH-specific therapy in CHD patients from the Netherlands and Singapore.

## Methods

### Study population

In this multicentre study, all adult CHD patients receiving PAH-specific therapy and enrolled in two nationwide registries between 2004 and 2013, using uniform inclusion criteria, were included. Dutch patients were participants of a prospective multicentre study on the effects of bosentan (ERA) therapy in PAH-CHD in the Netherlands [2, 4]. The patients from Singapore were enrolled in a dedicated PAH-CHD registry of the National University Hospital in Singapore at the start of PAH-specific therapy. PAH was diagnosed according to the guidelines in all patients [5].

In the Netherlands, all patients were started on treatment with bosentan, as part of the prospective multicentre study mentioned previously. In Singapore, the type of PAH treatment was decided by the treating physician, taking into account medical insurance policies. In both countries, timing to the start of combination therapy was at the discretion of the treating physician, considering patient preferences and existing contraindications. Sildenafil was commonly started at 20 mg three times daily and tadalafil at 20 mg once daily, increasing to 40 mg once daily. Bosentan monotherapy was started at 62.5 mg twice daily, increasing the dose to 125 mg twice daily after four weeks, as tolerated.

### Data collection and outcome definition

Exercise capacity was evaluated using the six-minute walk test (6MWT) and cardiopulmonary exercise test (CPET). Transthoracic echocardiography was performed following the available guidelines in both centres [5]. Patients with Down syndrome were excluded from all analyses, due to previous reports indicating that the 6MWT is not a valid indicator of cardiorespiratory fitness in these patients [16].

Primary outcome was improvement in 6MWT during follow-up. To accommodate for expected differences in height and weight between the two populations, measurements of the 6MWT were calculated as percentage of the predicted value, using the equation of Enright and Sherrill [17]. Improvement in 6MWT was defined as at least 10 % increase in 6MWT distance from baseline. Other parameters studied were peak VO_2_ at baseline and NYHA functional class and echocardiographic parameters at baseline and follow-up.

### Statistical analysis

Data are summarised as number (%) or mean ± SD as appropriate. Categorical data were evaluated using the chi-square statistic. Independent sample t-test was used for comparison of continuous variables. A linear mixed model with an autoregressive residual covariance matrix and a random intercept for individual differences was used to analyse 6MWT values during follow-up. Kaplan-Meier curves were plotted and log-rank test was performed to assess differences in the occurrence of mortality between the two cohorts. The relation between determinants and mortality was assessed using univariate and multivariate Cox proportional hazard analyses. Significant univariate determinants (entry threshold *p* = 0.10) were entered in a multivariate model analysed with a backward conditional algorithm. All reported p values are two-sided, and values of *p* < 0.05 were considered significant. Data analysis was performed using SPSS 20.0 (IBM).

## Results

### Patient cohort

**Fig. 1:**
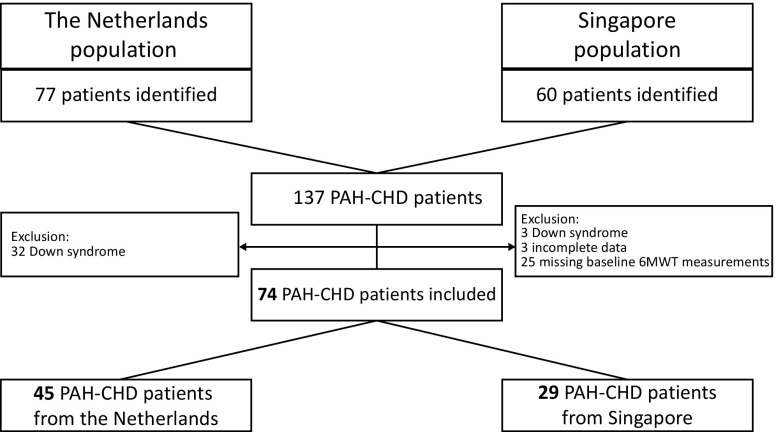
Derivation of the study population

A total of 74 PAH-CHD patients (29 Singaporean, 45 Dutch, mean age 45 ± 14 years, 31 % male) were analysed in this study (Fig. 1). Both populations were comparable regarding age, sex and mean duration of follow-up (Table 1), except for height (168 vs. 158 cm, *p* < 0.01) and weight (64 vs. 57 kg, *p* = 0.04).

**Table 1:** Baseline clinical characteristics

				All patients	The Netherlands	Singapore	
				(*n* = 74)	(*n* = 45)	(*n* = 29)	*p* value
Age (years)				45 ± 14	47 ± 14	41 ± 14	0.11
Male sex, n (%)				23 (31)	14 (31)	9 (31)	0.99
Follow up (years)				3.7 ± 2.4	3.3 ± 2.2	4.4 ± 2.8	0.06
Height (cm)				163 ± 10	168 ± 8	158 ± 9	<**0.01**
Weight (kg)				61 ± 15	64 ± 14	57 ± 15	**0.04**
Body surface area (m2)				1.7 ± 0.2	1.7 ± 0.2	1.6 ± 0.2	<**0.01**
Eisenmenger syndrome, n (%)				57 (77)	32 (71)	25 (86)	0.13
Pretricuspid shunt, n (%)				33 (45)	16 (36)	17 (59)	**0.05**
Posttricuspid shunt, n (%)				41 (55)	29 (64)	12 (41)	
Shunt closed, n (%)				4 (6)	2 (5)	2 (7)	0.67
Exercise capacity							
NYHA functional class III, n (%)				55 (74)	32 (71)	23 (79)	0.36
peak VO_2_ (ml/min)				792 ± 344	970 ± 357	660 ± 275	<**0.01**
peak VO_2_ (% of predicted)				41 ± 16	49 ± 18	35 ± 11	**0.01**
Baseline 6MWT (m)				363 ± 136	395 ± 137	312 ± 121	**0.01**
Baseline 6MWT (% of predicted)				62 ± 24	68 ± 25	53 ± 21	**0.02**
Echocardiography							
sPAP (mmHg)				84 ± 35	76 ± 32	99 ± 37	**0.01**
Impaired RV function, n (%)				17 (17)	8 (23)	9 (27)	0.67
Impaired LV function, n (%)				5 (5)	4 (11)	1 (3)	0.19
PAH medication, n (%)							
ERA				45 (61)	45 (100)	0	<**0.01**
PDE-5 inhibitor				29 (39)	0	29 (100)	<**0.01**
Combination therapy initiated				11 (15)	8 (18)	3 (10)	0.24
Discontinued PAH therapy				7 (10)	7 (16)	0	**0.03**

Values are presented as mean ± SD or as counts with percentages.

*NYHA* New York Heart Association, *peak VO*_2_ maximum oxygen consumption, *6MWT* six-minute walk test, *sPAP* systolic pulmonary arterial pressure, *RV* right ventricular, *LV* left ventricular, *PAH* pulmonary arterial hypertension, *ERA* endothelin receptor antagonist, *PDE-5* phosphodiesterase type 5.

All Dutch patients were started on the ERA bosentan, while the entire Singaporean population received a PDE-5 inhibitor (either sildenafil or tadalafil) as PAH treatment (Table 1). During follow-up, combination therapy consisting of adding an ERA or a PDE-5 inhibitor, was started in 18 % of the patients from the Netherlands and in 10 % of the Singapore patients (p = NS). In the course of the study 7 Dutch patients (16 %) discontinued their PAH-specific therapy. This was either due to amelioration of the pulmonary arterial pressures, patient preferences or the decision of the treating physician.

### Exercise capacity

Exercise capacity was significantly different between the two groups (Table 1). The peak VO_2_ measured by CPET was higher in the Dutch population, both the absolute peak VO_2_ value (970 ± 357 vs. 660 ± 275 ml/min, *p* < 0.01) and the percentage of predicted peak VO_2_ (49 ± 18 vs. 42 ± 13 %, *p* = 0.01). The 6MWT showed similar differences between the two populations at baseline. Dutch patients were able to walk an average of 395 meters during 6MWT, versus a mean of 312 meters for the Singaporean patients (*p* = 0.01). This difference persisted after correction for sex, height and weight, which was used to calculate an expected 6MWT value for each individual patient (68 vs. 53 % of predicted 6MWT, *p* = 0.02).

### Clinical outcome

**Fig. 2:**
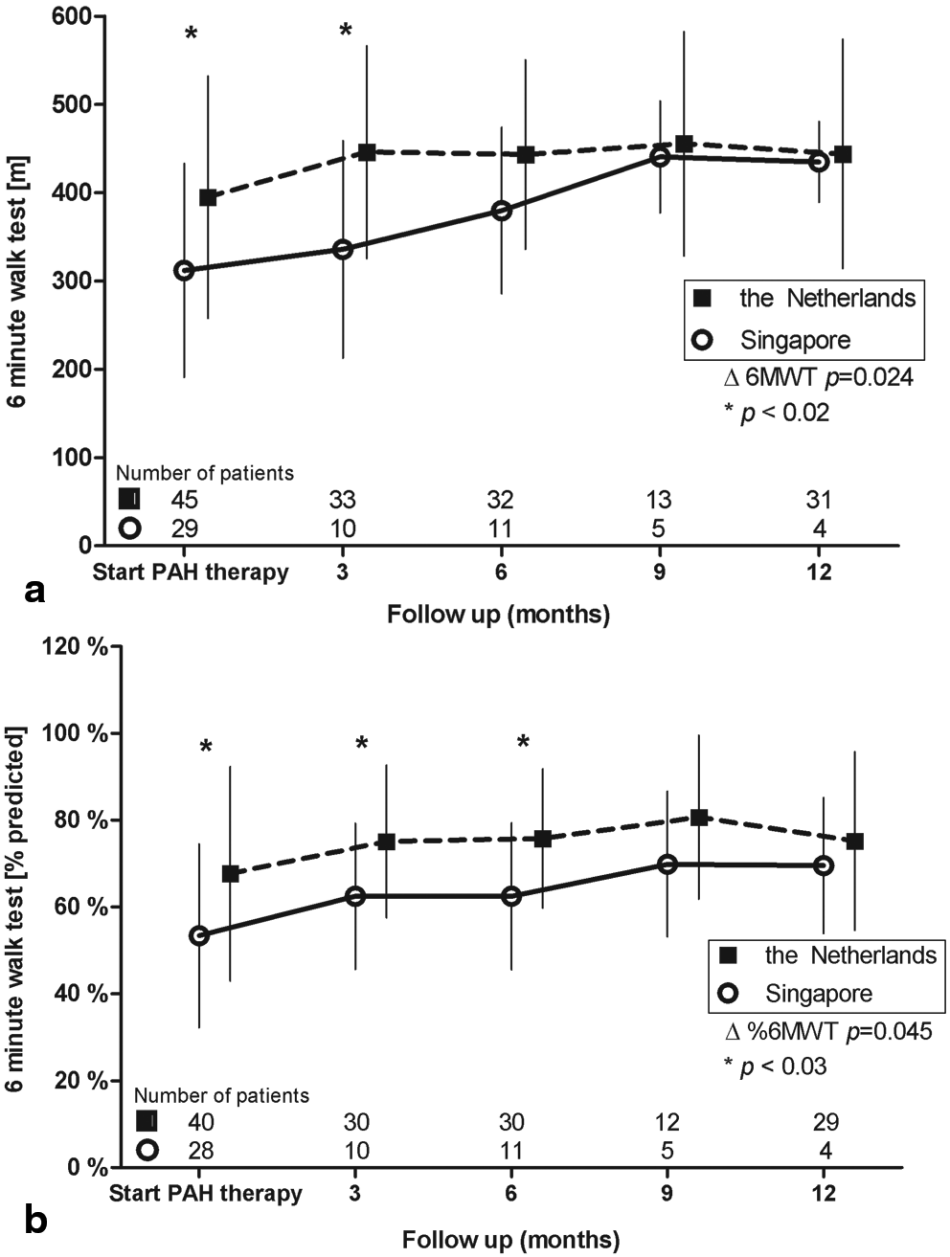
Change in six-minute walk test (6MWT) **a** 6MWT during follow-up, absolute values, **b** 6MWT expressed as percentage of predicted distance, corrected for sex, height and weight ∆6MWT = change in 6MWT from baseline to 12-month follow-up between Singaporean and Dutch patients

All CHD patients were regularly monitored by means of 6MWT measurements during the treatment of PAH. Figure 2a portrays the change in 6MWT distance during the first year of treatment in both cohorts. There was a clear and significant difference between the populations in 6MWT distance before the start of PAH-specific therapy, as noted previously, which persisted until the third month of treatment (*p* < 0.02 for both). The Singaporean patients showed a significantly higher increase in 6MWT distance, compared with the population from the Netherlands (*p* = 0.024). Figure 2b depicts the 6MWT distance expressed as a percentage of predicted. The differences between the two groups at baseline and three months remain, and even extend to the sixth month (*p* < 0.03 for all). Furthermore, besides the lower starting point for Singaporean patients, there was a significant difference in the change of 6MWT distance during 12 months of treatment in both groups, when corrected for percentage of predicted values (*p* = 0.045). Of note, the 6MWT measurements depicted in Fig. 2 are of survivors only, and the number of patients gradually decreases over time. Improvement in 6MWT was not significantly associated with an improvement in NYHA functional class or right ventricular function measured by echocardiography.

**Fig. 3:**
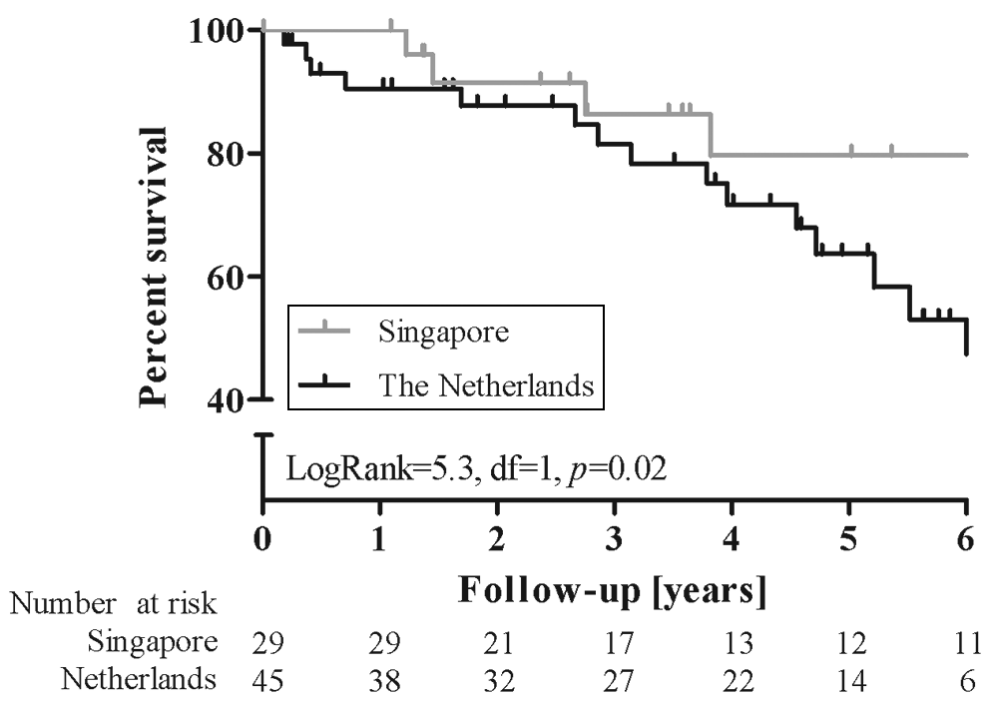
Kaplan-Meier curve estimates of overall survival according to country of origin

Surprisingly, survival was significantly lower in the Dutch population, as depicted by Kaplan-Meier analysis in Fig. 3 (log rank = 5.3, *p* = 0.02). However, when determining the relation between important clinical variables and mortality using multivariate Cox regression analysis, age at the start of advanced therapy was the most important predictor of mortality with a hazard ratio of 1.5 per 10 years of increasing age (95 % CI 1.1–2.1, *p* = 0.02) corrected for ethnicity, baseline 6MWT, sex and right ventricular function at baseline.

### Determinants of change in six-minute walk distance

**Fig. 4:**
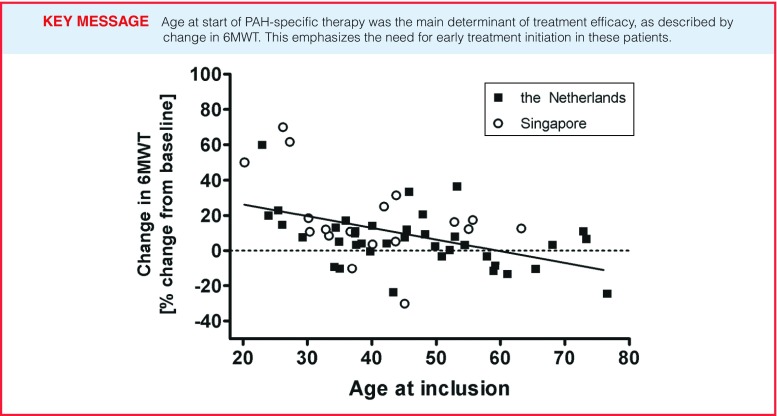
Change in six minute walk test (6MWT) per age at point of inclusion (start PAH-specific therapy)

Using mixed model linear regression, age at start of therapy (per 5 years lower age) was found to be a significant determinant of change in six-minute walk distance (Table 2), corrected for baseline 6MWT, sex, ethnicity and CHD defect (β= + 4.5, *p* = 0.017). We performed a sensitivity analysis to assess the model performance when excluding outliers (top or bottom 5 %), which yielded the same predictor of treatment efficacy: age at start of therapy (β = +2.3, *p* = 0.027). The effect of age at the start of therapy is further illustrated in Fig. 4, where it is clear that from the age of 45 the benefit of PAH-specific treatment on exercise capacity in both populations starts to approach zero.

**Table 2:** Regression coefficients for determinants of percentage change of 6MWT from baseline

				β	95 % CI	*p* value
Female sex				4.0	− 14.1–22.2	0.66
Singaporean ethnicity				7.4	− 13.2–27.9	0.48
Age at start advanced therapy (per 5 year lower)				4.5	0.8–8.1	**0.017**
Pretricuspid shunt				0.7	− 18.8–20.1	0.95
PDE-5 inhibitor treatment				7.4	− 13.2–27.9	0.48

*6MWT* six-minute walk test, *PDE5* phosphodiesterase type 5.

## Discussion

This study elucidates the variations in clinical presentation and outcome of two CHD populations receiving PAH-specific therapy. While Singaporean patients had a significantly lower exercise capacity before treatment initiation, exercise capacity increased equally in both cohorts, despite a clear difference in choice of therapy. Furthermore, age at initiation of PAH-specific therapy was shown to be the strongest determinant of treatment effect, when corrected for ethnicity, sex, exercise capacity at baseline and CHD defect. This underlines the need for starting treatment early in these patients.

### Global differences

There are several potential causes for the difference in exercise capacity before treatment initiation, which can be explained by several factors and not only by a difference in ethnicity itself. One explanation is the higher number - although not statistically significant—of Eisenmenger patients in the Singapore population. This could represent a more advanced disease state at the start of PAH-specific therapy, which is known to be associated with decreased exercise capacity [18, 19]. Furthermore, the anticipated difference in social economic status between the studied Dutch and Singaporean patients can explain the baseline differences even more. As described by Wu et al. [15] PAH patients with a lower socioeconomic status have an increased risk of clinical worsening compared with patients with a higher socioeconomic status. One of the explanations for this difference is that PAH-targeted therapies impose a tremendous economic burden on Singaporean patients as they are not covered by insurance. While all residents from Singapore have compulsory basic medical insurance, only a small number have extended private medical insurance (28 % in our cohort). These financial barriers may limit patient access to health services and appropriate treatment, imposing a disproportionate burden on those with a lower socioeconomic status. This is further facilitated by pulmonary hypertension not being listed as one of the chronic conditions that receive treatment reimbursement in Singapore [20]. This also explains the difference in choice for PAH-specific treatment regimen, with the annual cost of sildenafil around $ 3300–$ 5500 versus ≈$ 36,700 for bosentan [15]. In the Netherlands, health insurance is also compulsory; however, both primary and hospital care is accessible for CHD patients without extra costs [21]. This is of clinical relevance, since quality of life in PAH-CHD patients has recently been associated with worse outcome [22, 23].

### Effect of treatment on functional capacity

Previous results have reported the important effects of age on single exercise capacity measurements in CHD patients [24, 25]. In CHD patients with PAH a decreased exercise capacity is often present, due to the inability to increase cardiac output sufficiently to meet increased demand [26]. During ageing, the loss of RV contractile reserve and increasing pulmonary dysfunction, combined with possible diastolic dysfunction of the left ventricle, further impairs the ability to increase cardiac output in these patients [27]. Additionally, while the short-term prognosis of PAH-CHD patients is often good, a markedly increased morbidity and mortality has been shown with increasing patient age at the time of diagnosis [28]. In addition to a diminished exercise capacity inherent to older age, we were now able to show that age has a significant negative influence on mortality and the ability to improve exercise capacity during PAH treatment. An explanation for the lack in improvement might be that PAH therapy antagonises the natural declining exercise capacity in older patients, leading to stabilisation of 6MWT rather than improvement. Furthermore, the disease of the pulmonary vasculature could be more advanced in patients presenting at older age, thereby limiting the ability in these patients to improve in functional capacity. An early initiation of combination therapy might be beneficial in these older patients with regard to exercise capacity and possibly survival [29]. However, due to the retrospective nature of the study, we were unable to show if an earlier intervention would have prevented a decline in 6MWT in older patients.

Because CPET is a demanding test for patients and technicians, the relatively simple 6MWT has frequently been used as a clinical endpoint and routine follow-up measurement for CHD-PAH patients. Indeed improvement of 6MWT is a valuable goal in these patients and reduces mortality and morbidity in the short term [2, 4]. However, meta-analysis has shown that changes in 6MWT do not predict long-term clinical events [30]. Therefore, future clinical studies should certainly focus on clinical worsening as a more meaningful endpoint.

### Limitations

In this registry-based study we could not control exposure or outcome assessment, but instead needed to rely on others for accurate record-keeping. This could have introduced selection bias in the patient cohort and measurements during follow-up. Furthermore the population used in this study reflects the clinical and research workload of tertiary CHD centres, possibly introducing bias by favouring those with more symptoms and lower perceived functional capacity.

## Conclusion

Patients from Singapore had a worse clinical performance at baseline compared with the PAH-CHD patients from the Netherlands. Age at initiation of PAH-specific therapy was the strongest predictor of treatment efficacy and mortality, emphasising the need for early initiation of treatment in these patients.
